# Determination of Cell Viability—Trypan Blue Staining

**DOI:** 10.1111/cpr.70209

**Published:** 2026-05-12

**Authors:** Yingying Liu, Wenfeng Huang, Boqiang Fu, Jing Wang, Lei Wang, Jiani Cao, Jie Hao, Tongbiao Zhao, Ranran Xu, Aijin Ma, Kai Chen

**Affiliations:** ^1^ National Institute of Metrology Beijing China; ^2^ Standards Committee, Chinese Society for Cell Biology Shanghai China; ^3^ Institute of Zoology Chinese Academy of Sciences Beijing China; ^4^ Beijing Institute for Stem Cell and Regenerative Medicine Beijing China; ^5^ Zephyrm Biotechnologies Co. Ltd. Beijing China; ^6^ Beijing Technology and Business University Beijing China; ^7^ Shanghai RuiYu Biotech Co. Ltd. Shanghai China

Cell viability measurement is a routine quality control test conducted in the biotechnology industry, such as regenerative medicine and immunotherapy. The standard ‘Determination of cell viability—Trypan blue staining’ specifies the method for measuring cell viability by trypan blue staining, including principle, reagents or materials, instruments, sample preparation, measurement procedure, result calculation, method validation and result report. It is applicable to cell research and production. This standard is jointly drafted and agreed upon by experts from the Standard Committee of Chinese Society for Cell Biology and was first released on 28 October 2024. We hope that the publication of this standard document will promote the data consistency and comparability of cell viability measurements across different laboratories, platforms and operators.

## 1 Scope

This document specifies the method for measuring cell viability by trypan blue staining, including principle, reagents and materials, equipment, sample preparation, measurement procedure, result calculation, method validation and result report.

This document is applicable to cell viability measurements in monodisperse cell suspensions derived from a subculture of mammalian cell lines.

This document is not applicable to mammalian cells resuscitated from cryopreservation or directly dissociated from primary tissues.

## 2 Terms and Definitions

### 2.1 Cell Counting

Measurement process to determining the cell number.

### 2.2 Cell Viability

The proportion of live cells in the total cell population.

## 3 Principle

Trypan blue is an azo, hydrophilic, acidic blue dye that can penetrate the cell membranes of dead cells and dying cells, whereas viable cells can exclude trypan blue due to the integrity of their cell membranes. Based on this principle, viable cells (which appear transparent and colourless) and dead cells (which appear blue) can be distinguished by their colour difference under microscope imaging. The cell viability is determined by calculating the percentage ratio of viable cells to the total number of cells within the same measured area.

## 4 Reagents and Materials

### 4.1 Trypan Blue Dye or Trypan Blue Staining Solution

Trypan blue dye shall be chemically pure. The concentration of trypan blue staining solution shall be 0.2% ~ 0.4%.

### 4.2 Buffer Solution

The buffer solution shall not cause rupture, significant morphological changes, or clustering of the cells to be tested. A phosphate buffer without Ca^2+^ or Mg^2 +^ can be used.

### 4.3 100 mL Volumetric Flask Can Be Used

The pore size of cell filter should be between 40 and 100 μm.

### 4.4 Cell Counting Chamber

The cell counting chamber shall be clean and free of cosmetic marks, stains and other defects.

## 5 Instruments

### 5.1 Light Microscope or Cell Counter

The light microscope shall have a bright field with either a ×10 or ×20 objective.

The cell counter shall include a trypan blue mode.

### 5.2 Electronic Balance

Readability (resolution) shall be 1 mg.

### 5.3 Centrifuge

Rotational speed range: 500 to 15,000 r/min.

### 5.4 Vortex Oscillator

Rotational speed range: 0 to 3000 r/min.

### 5.5 Micropipette

Volume ranges: 0 to 20, 0 to 200 μL and 0 to 1000 μL.

The micropipettes shall be calibrated to meet the test requirements.

## 6 Sample Preparation

Cells in the tested samples shall be uniformly dispersed as individuals and processed to obtain a monodisperse cell suspension before analysis.

A cell filter with a pore size of 40 to 100 μm should be used to meet the requirements.

Staining and measurements shall be performed immediately after sample collection and preparation.

## 7 Measurement Procedure

### 7.1 Trypan Blue Staining Solution Preparation

Accurately weigh 0.2 to 0.4 g of trypan blue dye and add 60 mL of buffer solution to fully dissolve the dye. Then, adjust the volume of trypan blue staining solution to 100 mL using the buffer. The trypan blue solution can be left at room temperature for 1 day. Afterwards, the solution shall be centrifuged at 15,000 r/min for 5 to 10 min to remove any precipitate, and the supernatant can be retained for use.

Commercially available 0.2% to 0.4% trypan blue staining solutions can also be used.

### 7.2 Sample Dilution

Microscopic measurements: Dilute the sample cell concentrations using a buffer solution to 1 × 10^6^/mL to 2 × 10^6^/mL.

Cell counter measurement: Dilute the sample cell concentrations according to the method and concentration range recommended by the instrument manufacturer.

### 7.3 Sample Staining

The diluted cell suspension shall be thoroughly mixed and stained with a 0.2% to 0.4% trypan blue staining solution at a 1:1 ratio at room temperature. The recommended staining duration is 2 to 10 min, or it can be adjusted and optimised according to the specific cell type being measured.

### 7.4 Performing Measurement

Light microscope measurement: After the stained cell samples, as described in Section 7.3, are thoroughly mixed, add 10 to 20 μL of the solution to the cell counting chamber with a scale counting grid. After resting for about 1 min, count the cells under the microscope using a low‐power objective (×10 or ×20). Record the number of blue cells (damaged and dead cells) and transparent, colourless cells (viable cells) separately. The total number of counted cells in a single measurement shall exceed 300.

Cell counter measurement: After the stained cell samples, as described in Section 7.3, are thoroughly mixed, add 10 to 20 μL of the solution to the cell counting chamber specifically designed for the cell counter being used. After resting for about 1 min, insert the cell counting chamber into the cell counter, select the trypan blue mode, and set the dilution ratio to 1:1 to measure the cell count and cell viability.

To improve the accuracy and reproducibility of cell activity measurements, it is recommended to perform at least three measurements of parallel samples.

## 8 Result Calculation

Calculate the cell viability according to formula (1):

(1) $$P=\frac{N_{h}}{N_{s}+N_{h}}\times 100\%$$

Cell viability *N*_*h*_ *‐*the number of transparent viable cells *N*_*s*_ *‐*the number of dead cells in blue.

## 9 Method Validation

Method performance shall be validated before applying the method to a specific cell sample. Key performance parameters include accuracy, repeatability precision, method robustness and reproducibility precision, which encompass inter‐operator, inter‐instrument and inter‐day variation.

Scheme and standards for evaluating method performance parameters shall be established, and corresponding documents shall be developed and saved

## 10 Record and Report

Record the test conditions, measurement results and calculation results. The information provided in the test record and result report shall include at least:

- sample information;
- instrument information;
- name, brand name and batch number of the reagent;
- measurement result;
- data analysis procedure;
- remarks.

Record and report templates can refer to Appendix A.

## Author Contributions

Liu Yingying, Fu Boqiang and Wang Jing contributed to the conception and design. Liu Yingying, Huang Wenfeng and Chen Kai performed the validation experiment. Liu Yingying and Fu Boqiang drafted and revised the manuscript. Wang Lei, Cao Jian, Hao Jie, Zhao Tongbiao, Xu Ranran and Ma Aijin critically read and revised the manuscript.

## Funding

This work was supported by the National Key Research and Development Program of China (2023YFF0613600).

## Data Availability

The data that support the findings of this study are available from the corresponding author upon reasonable request.

## Appendix A

Cell viability measurement by trypan blue staining test record/results report.

